# Entropy, Carnot Cycle, and Information Theory

**DOI:** 10.3390/e21010003

**Published:** 2018-12-20

**Authors:** Mario Martinelli

**Affiliations:** Dipartimento di Elettronica Informazione e Bioingegneria, Politecnico di Milano, 20133 Milano, Italy; mario.martinelli@polimi.it

**Keywords:** entropy, Carnot cycle, information theory, Kullback–Leibler divergence

## Abstract

The fundamental intuition that Carnot had in analyzing the operation of steam machines is that something remains constant during the reversible thermodynamic cycle. This invariant quantity was later named “entropy” by Clausius. Jaynes proposed a unitary view of thermodynamics and information theory based on statistical thermodynamics. The unitary vision allows us to analyze the Carnot cycle and to study what happens when the entropy between the beginning and end of the isothermal expansion of the cycle is considered. It is shown that, in connection with a non-zero Kullback–Leibler distance, minor free-energy is available from the cycle. Moreover, the analysis of the adiabatic part of the cycle shows that the internal conversion between energy and work is perturbed by the cost introduced by the code conversion. In summary, the information theoretical tools could help to better understand some details of the cycle and the origin of possible asymmetries.

## 1. Carnot Cycle and Thermodynamics

Since its origin, information theory has correlated with thermodynamics [1]. Shannon [2], who mentioned in his fundamental work of formulating the 2nd theorem, admitted that: “The form H will be recognized as that of entropy as defined in certain formulation of statistical mechanics”. It is Jaynes; however, that pointed out the close relationship between thermodynamics and information theory [3,4], which shares the concept of “partition function”: “…the basic mathematical identity of the two fields [thermodynamics and information theory] has had, thus far, very little influence on the development of either. There is an inevitable difference in detail, because the applications are so different; but we should at least develop a certain area of common language…we suggest that one way of doing this is to recognize that the partition function, for many decades the standard avenue through which calculations in statistical mechanics are “channeled”, is equally fundamental to communication theory”.

The partition function *Z*(*λ*) connects the thermodynamics variables to the statistics of the process [5] through the two constitutive expressions shown as follows:

(1) $$\langle f\rangle =-\frac{d}{d\lambda }lnZ\left(\lambda \right)$$

(2) S=klnZ(λ)+ kλ〈f〉

where *k* is the Boltzmann constant, *λ* is the Lagrange multiplier (in thermodynamics formalism equivalent to *1/kT*), and 〈f〉 is the expectation value of generic function of random variables belonging to the analyzed process. In thermodynamics, this function is the internal energy *U*, while in information theory, it is the code-word length *l.* In Equation (2) the entropy, the “measure of the disorder” is

(3) $$S=-k\sum_{i}p_{i}lnp_{i}$$

where *p_i_* is the probability of finding the system in the ith state. The “Helmholtz potential” or Free-Energy *F* is by definition

(4) $$F=-\frac{1}{\lambda }lnZ$$

hence, Equation (2) can be also expressed in the standard thermodynamics formalism as

(5) $$S=-k\lambda F+\;k\lambda \langle U\rangle =-\frac{F}{T}+\frac{\langle U\rangle }{T}$$

Often, in information theory, the Entropy is expressed by putting equal to unity the Boltzmann constant [6]. In this case, Equation (2) becomes [3]

(6) S=lnZ(λ)+ λ〈l〉

Equations (1) and (2) are so important for the thermodynamics that Schrodinger said [7]: “…it may be said to contain, in a nutshell, the whole of thermodynamics which hinges entirely on this basic distribution”.

However, in 1959, Jaynes [4] pointed out some “singularities” of the information theory that are not found in thermodynamics and; therefore, prevent the development of simple parallelism. In particular, Jaynes noted that the Shannon’s 1st theorem, which defines the channel capacity, does not present an equivalent of channel capacity in thermodynamics. 

The situation has been summarized recently in an important paper by Merhav [8], who says: “While the laws of physics draw the boundaries between the possible and impossible in Nature, the coding theorems of information theory together with their converse parts, draws the boundaries between the possible and impossible in digital communications.” 

The above scenario inhibits extension of all the fundamental axioms of the thermodynamics to information theory, including the Carnot theorem that, in consequence, does not present an exact parallel in information theory.

As clearly pointed out by Cardoso Diaz [9], the Carnot’s principle is “a principle on the possibility of recovery of the initial condition of operation, so that a thermal engine can start a new cycle of operations “. If we apply the same principle to a communication process (intended here as suggested by Reiss [10]: “the process of communication in which information is transmitted from a source ‘system’ to a receiving ‘environment’”) in a context of “small dimensionality” (small alphabet or limited number of words), which situation can perturb this symmetry? The aim of this paper is to explore this scenario.

**Figure 1:**
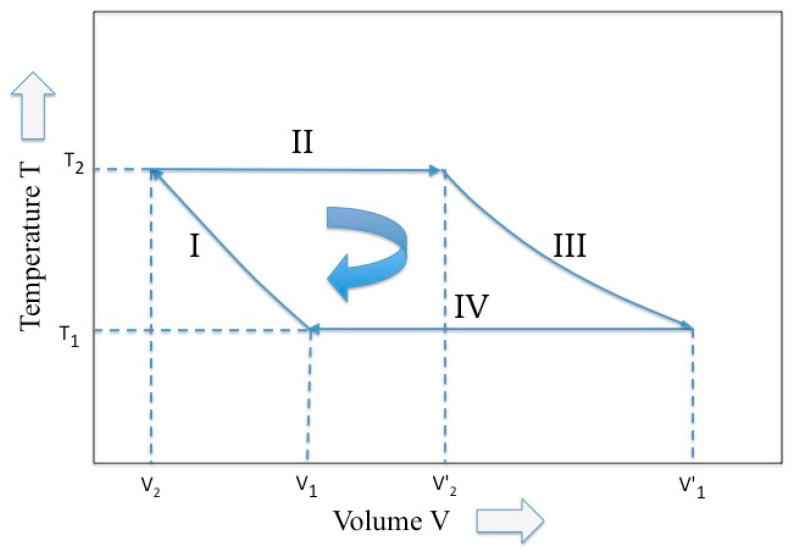
The Carnot cycle described by two variables, temperature and volume (elaborated from Reference [10]).

Among a great variety of Carnot cycle representation, the representation proposed by Max Planck in his treatise of thermodynamics [11] is used (see Figure 1), where two variables, temperature and volume, are picked up. Following the Planck notation, we have that, in the four phases of the Carnot cycle and for a perfect gas (i.e., adiabatic compression, isothermal expansion, adiabatic expansion, and isothermal compression), the external work *W* is given by:

(7) $$W=-\int_{v_{1},T_{1}}^{v_{2},T_{2}}PdV-\int_{v_{2},T_{2}}^{v_{2}^{\prime },T_{2}}PdV-\int_{v_{2}^{\prime },T_{2}}^{v_{1}^{\prime },T_{1}}PdV-\int_{v_{1}^{\prime },T_{1}}^{v_{1},T_{1}}PdV\;$$

where *P* is the pressure and *V* is the volume. If considering a single mole of perfect gas and by introducing the gas at a constant volume specific heat *c_v_*, the (7) becomes

(8) $$W=c_{v}\left(T_{2}-T_{1}\right)-kT_{2}ln\left(\frac{V_{2}^{\prime }}{V_{2}}\right)+c_{v}\left(T_{1}-T_{2}\right)-kT_{1}ln\left(\frac{V_{1}}{V_{1}^{\prime }}\right)$$

This equation points out that the net work production is possible, provided that two reservoirs are available at the temperatures *T*_2_ and *T*_1_. In fact, although the first and third terms in Equations (8) self-cancel, the second term prevails on the fourth and a net work is hence generated after the cycle. 

Since “the work acquired (delivered) in a reversible isothermal process is equal to the increase (decrease) of the Helmholtz potential” [11], we may write the second term in Equation (8) as:

(9) $$\Delta F_{2}=-kT_{2}ln\left(\frac{V_{2}^{\prime }}{V_{2}}\right)$$

where *F* is the Helmholtz potential. This net work is equal to the amount of the direct heat transfer from the high-temperature reservoir to the gas of the piston. 

In correspondence of this change in the Helmholtz potential and for the reversible case, the entropy of the system increases by the quantity:

(10) $$\Delta S_{2}=-\frac{\Delta F_{2}}{T_{2}}=kln\left(\frac{V_{2}^{\prime }}{V_{2}}\right)$$

Similarly, it can be written for the fourth phase of the cycle. Therefore, the entropy of the whole cycle is conserved if

(11) $$kln\left(\frac{V_{2}^{\prime }}{V_{2}}\right)=kln\left(\frac{V_{1}}{V_{1}^{\prime }}\right)=-kln\left(\frac{V_{1}^{\prime }}{V_{1}}\right)$$

or

(12) $$\left(\frac{V_{2}^{\prime }}{V_{2}}\right)=\left(\frac{V_{1}^{\prime }}{V_{1}}\right)$$

Equations (11) and (12) summarize the effect of the entropy conservation on the Carnot cycle. In other words, the ratio between the final and initial volume experimented by the gas during the reversible isothermal expansion phase must be maintained even during the reversible compression phase, even at different temperatures. This relationship was already pointed out by Planck in his treatise [11].

The first and third terms in Equations (8) represent the work given by the external work reservoir on the system and the work given by the system on the external work reservoir, respectively. These two terms have an opposite sign and the total work provided by the adiabatic cycles on the system is hence zero [12]. In an adiabatic process, the entropy of the phase does not change and the work has been produced only at the expense of the internal energy. Hence, we have that

(13) $$\Delta U=-c_{v}\left(T_{2}-T_{1}\right)$$

The entropy expression for a perfect gas in the adiabatic process is written as:

(14) $$dS=c_{v}\frac{dT}{T}+k\frac{dV}{V}$$

when the change of entropy is zero, Equation (19) can be described as:

(15) $$\frac{dT}{T}=-\frac{k}{c_{v}}\frac{dV}{V}$$

**Figure 2:**
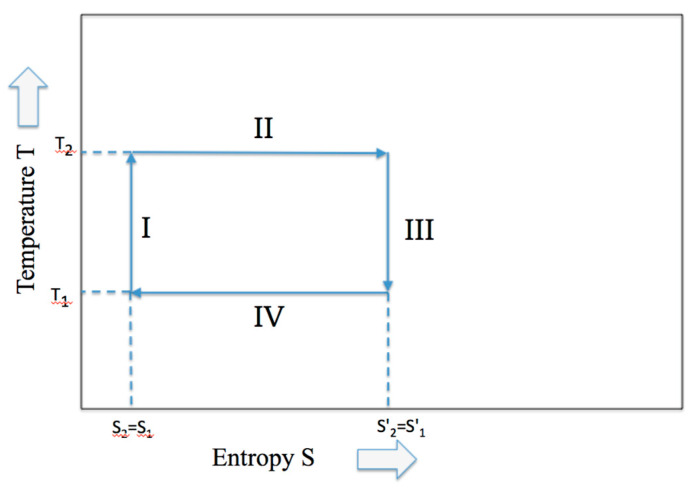
The Carnot cycle described by the two variables, temperature and entropy.

During the adiabatic expansion, the increase of entropy due to the volume increase of the piston expansion is compensated by an equivalent decrease of internal energy connected to an equivalent of the temperature decrease. Hence, although the entropy remains constant under the adiabatic condition, the work is produced at the expense of the internal energy (similarly, the increase of internal energy is at the expense of work for the adiabatic compression). In Figure 2 the classical Temperature-Entropy representation of the Carnot cycle is given.

## 2. Carnot Cycle and Information Theory

During the isothermal expansion (i.e., consider phase II) the flux of energy coming from the high-temperature reservoir is converted only in free-energy, because of Equation (9), thus is useful in this work. Hence, according to Equation (2) the change in entropy is

(16) $$S_{2}^{\prime }-S_{2}=klnZ_{2}^{\prime }\left(\lambda_{2}\right)-klnZ_{2}\left(\lambda_{2}\right)$$

where the two partition functions $Z_{2}^{\prime }$ and $Z_{2}$ share the same statistics $p_{i}$ but different eigenvalues of energy (i.e., $\eta_{i}$ and $\varepsilon_{i}$)

(17) $$Z_{2}^{\prime }=\sum_{i}e^{-\lambda_{2}\eta_{i}}\;\mathrm{and}\;Z_{2}=\sum_{i}e^{-\lambda_{2}\varepsilon_{i}}$$

In fact, according to Tribus [12] and Figure 1 therein, “a change of work induces only a change in the value of the energy levels, not in the statistics”. If we compare Equations (16) and (10) we observe that during the isothermal expansion the partition function and the volume play the same role in giving the change of entropy.

Let us suppose now that a perturbation occurs that changes the final distribution, which refers to the same ensemble but with terms *q_i_*. This perturbation affects the final partition function, which becomes now

(18) $$Z_{2}^{\prime \prime }=\sum_{i}e^{-\lambda_{2}\theta_{i}}$$

where $\theta_{i}$ are the new energy eigenvalues. The distance between the final and initial distribution will be measured by the appropriate Kullback–Leibler [6,13] divergence and will be

(19) $$kD\left(q∥p\right)=k\underset{i}{\sum }q_{i}ln\frac{q_{i}}{p_{i}}=k\underset{i}{\sum }q_{i}ln\frac{\frac{e^{-\lambda_{2}\theta_{i}}}{Z_{2}^{\prime \prime }}}{\frac{e^{-\lambda_{2}\eta_{i}}}{Z_{2}^{\prime }}}=k\lambda_{2}\underset{i}{\sum }q_{i}\left(-\theta_{i}+\eta_{i}\right)-klnZ_{2}^{\prime \prime }+klnZ_{2}^{\prime }$$

Because of expression (4) we have

(20) $$k\lambda_{2}\Delta F_{2}=k\lambda_{2}\left(\;F_{2}^{\prime \prime }-F_{2}^{\prime }\right)=k\left(-lnZ_{2}^{\prime \prime }+lnZ_{2}^{\prime }\right)$$

and by introducing the average of the internal energy change due to the transformation (evaluated on the final distribution)

(21) $$\Delta U=\underset{i}{\sum }q_{i}\left(\theta_{i}-\eta_{i}\right)$$

we obtain that

(22) $$kD\left(q∥p\right)=k\lambda_{2}\Delta F_{2}-k\lambda_{2}\langle \Delta U_{2}\rangle$$

(we introduced the standard notation (6) for the Kullback–Leidler divergence). In a fundamental paper on the “thermodynamics of the decision”, Ortega and Braun [14] obtained a similar expression by using the expansion of the piston (and the relative change of volume) as an “archetypical” of an information process where the position of the piston modifies the knowledge about the initial state, described by an “a priori” distribution (*p_i_*) in a final state described by an “a posteriori” distribution (*q_i_*). According to this point of view, they describe Equation (22) as “…the free-energy difference consists of two terms: the average free-energy of the individual compartments (note: in [14] the enumeration of the states characterized by different energy labels are call “compartments”) and a cost term that measures the information theoretic distance between the initial and final information state, which is then converted into units of energy”. Moreover, they note that “While expression of the free-energy instantiates a trade-off between the internal energy 〈f〉 and the entropic cost S…we generalize these previous models of bounded rationality based on the duality between information and utility: Instead of considering absolute free-energy F we consider difference in free-energy ΔF between an initial state and a final state corresponding to the situation before and after the deliberation associated with the decision-making process”. Following this interpretation, the “variational free-energy principle” can be applied to different contexts: the perceptual decision making, the sensorimotor control, and so on [14].

If, instead of evaluating the distance between the final and initial distribution, we evaluate the vice-versa we obtain

(23) $$kD\left(p∥q\right)=k\underset{i}{\sum }p_{i}ln\frac{p_{i}}{q_{i}}=k\underset{i}{\sum }p_{i}ln\frac{\frac{e^{-\lambda_{2}\eta_{i}}}{Z_{2}^{\prime }}}{\frac{e^{-\lambda_{2}\theta_{i}}}{Z_{2}^{\prime \prime }}}=k\lambda_{2}\underset{i}{\sum }q_{i}\left(\theta_{i}-\eta_{i}\right)+klnZ_{2}^{\prime \prime }-\mathrm{kln}Z_{2}^{\prime }$$

or, by using the relationship (2)

(24) $$kD\left(p∥q\right)=S_{2}^{\prime \prime }-S_{2}^{\prime }$$

which points out that the KL divergence is “a measure of the inefficiency of assuming q_i_ as final distribution” (Cover [6]). The change of entropy of the isothermal expansion hence becomes

(25) $$S_{2}^{\prime }-S_{2}=S_{2}^{\prime \prime }-kD\left(p∥q\right)-S_{2}$$

Since the change of entropy in the isothermal cycle coincides with the given free-energy, expression of Equation (25) suggests that if during the expansion something occurred which modifies the final statistics, less free-energy is available, which is less work. In fact, according to Equation (23), part of this energy is converted in internal energy. Since the change in free-energy represents the minimum work 〈W〉 performed on the system in order to change its state, expression of Equation (25) suggests that this work is lowered by the mutual information term kD(p∥q) or

(26) 〈W〉≥ΔF− kD(p∥q)

**Figure 3:**
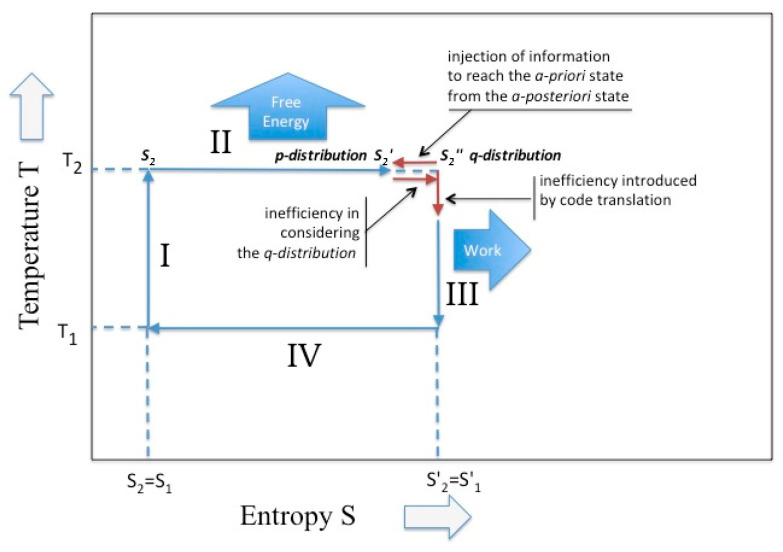
The Carnot cycle in the presence of intervention on the partition function. In the isothermal phase the existence of a Kullback–Leibler divergence; in the adiabatic phase the existence of an alphabet change.

Sagawa [15] obtain a similar expression while considering the case of a thermodynamics system maintained in non-equilibrium by means of a proper feed-back mechanism. In other papers [16,17] he proposes, for such systems, the term of “information engine”, because it converts information in energy (see Figure 3).

In the adiabatic phase of the Carnot cycle (i.e., the phase III and I in Figure 1) the situation appears more complex. According to Cardoso Diaz [9], the adiabatic phases are only functional to “recovery of the initial condition”. The two thermal reservoirs, which guarantee the reversible heat transference, are now replaced by the two work reservoirs (that, for an ideal gas, guarantee the reversible volume variation) and the temperature is allowed to pass. In general, the conditioning exerted by the bound work reservoir does not guarantee a unique course of the cycle. In fact, in discussing the adiabatic phase, Reiss [18] underlines that: “The performance of a definite amount of work does not necessarily guarantee arrival at a unique end state; whereas, arrival at a unique end state, starting from a unique initial state, seems to guarantee the performance of a definite amount of work”. In the Carnot cycle the adiabatic phases are ruled by the “gas laws “that permit to establish the final temperature on the basis of the yielded/absorbed work, through Equation (15). Since Carnot imposes the constancy of the entropy, the work is yielded/absorbed at the expenses of the internal energy. Hence, during the adiabatic phase, we observe a conversion of the entropy in internal energy and of this latter in work. This yields a dramatic change of the partition functions that are now expressed in terms of a “different ensemble” and “different temperature”. According to the expression of Equation (2), we have that (consider the phase III)

(27) $$S_{2}^{\prime }=klnZ_{2}^{\prime }\left(\lambda_{2}\right)=klnZ_{1}^{\prime }\left(\lambda_{1}^{\prime }\right)=S_{1}^{\prime }$$

or

(28) $$Z_{2}^{\prime }=\sum_{i}e^{-\lambda_{2}\eta_{i}}=\sum_{j}e^{-\lambda_{1}{ο}_{j}}=Z_{1}^{\prime }$$

where ${ο}_{j}$ are the new energy eigenvalues of the state $S_{1}^{\prime }$. On the other hand, in information theory, the temperature is connected with the inverse of the Lagrangian multiplier, whose maximum under the further constraint *Z* = 1 is related to the “maximum entropy of the distribution” (see Csiszar [19] and Jaynes [4]), given also by the logarithm of the vocabulary dimension *A* or by the logarithm of the alphabet dimension *A* (when any word is a symbol)

(29) $$\lambda_{max}=ln\left(A\right)$$

Hence, the change of temperature from *T_2_* to *T_1_* that occurs during the adiabatic cycle involves a change of the dimension of the ensemble on which the partition function is evaluated (in fact, the partition function upper limits results function of the temperature).

In order to gain comprehension, let us consider the total differential with respect to the temperature of the entropy function at the beginning of the adiabatic phase (for simplicity let us remove the index and apex of the function S)

(30) $$S=klnZ=kln\sum_{i=1}^{N\left(T\right)}e^{-\frac{\eta_{i}\left(T\right)}{kT}}$$

we have (by applying the Leibnitz rule)

(31) $$\begin{array}{cc} dS=k\frac{1}{Z}\left[\frac{d}{dT}Z\right]dT & =k\frac{1}{Z}[(\sum_{i=1}^{N\left(T\right)}\frac{\eta_{i}}{kT^{2}}e^{-\frac{\eta_{i}}{kT}})-\frac{1}{kT}\sum_{i=1}^{N\left(T\right)}\frac{d\eta_{1}}{dT}e^{-\frac{\eta_{i}\left(T\right)}{kT}}+e^{-\frac{\eta_{Up}}{kT}}\frac{dN\left(T\right)}{dT}]dT\\ & =\frac{1}{T^{2}}\left(\sum_{i=1}^{N\left(T\right)}p_{i}\eta_{i}\right)dT-\frac{1}{T}\sum_{i=1}^{N\left(T\right)}p_{i}d\eta_{i}+\frac{k}{Z}e^{-\frac{\eta_{Up}}{kT}}\frac{dN\left(T\right)}{dT}dT\\ & =\frac{U}{T}\frac{dT}{T}-\frac{dW}{T}+kp_{Up}dN\left(T\right) \end{array}\;$$

where $\eta_{Up}$ and $p_{Up}$ are, respectively, the energy eigenstate and the probability referring to the upper index of the sum. Hence, by imposing a constancy in the entropy (*dS* = 0) we have

(32) $$dW=\langle U\rangle \frac{dT}{T}+Tkp_{Up}dN\left(T\right)$$

that confirms that the work is produced at the expense of internal energy and change of the ensemble. In the case of a gas, the last term is *negligible* and we obtain

(33) $$dW=-PdV=-kT\frac{dV}{V}=\langle U\rangle \frac{dT}{T}$$

or

(34) $$\frac{dT}{T}=-\frac{kT}{U}\frac{dV}{V}$$

and being by definition *U/T = c_v_*, we re-obtain Equation (20). For small ensembles, typical of the information theory, the second term after the equality in Equation (32) cannot be neglected. In particular, since the upper limit of the sum is the dimension *A* (previously defined), if the maximum entropy $\lambda_{max}$ increases, *dN* increases. This means that less work is available for this phase of the cycle. Hence, the adiabatic phase realizes a sort of code-translation (re-arranging the dimension of the words in function of the new alphabet) that introduces “cost” in the energy-conversion (from internal energy to work). This result seems reasonable because any re-coding process will have a "cost", but its value will depend on the details of the process itself and must be the result of future investigations.

## 3. Comments and Conclusions

The reversible Carnot cycle realizes a balance of the entropy by the implementation of a quasi-symmetrical cycle, where the entropies of the two isothermal phases are balanced in value despite the difference in temperature. Moreover, the two adiabatic phases of the cycle play a role in realizing the temperature change. 

The increase of entropy due to the volume expansion of the thermodynamics vector (the ideal gas) generates ordered energy (the mechanical expansion of the piston), which yields net work only partially compensated by the work injected during the compression phase. Hence, the Carnot cycle is an excellent (the best) converter of disordered energy (the heat) into ordered energy (the piston work). Since the amount of available work is measured by the Helmholtz free-energy, we may also say that the Carnot cycle is an excellent converter of heat *Q* into free-energy *F*. 

However, the equivalence between thermodynamics parameters and information theoretical parameters begins to perturb this vision. Although equations (1) and (2) has been recovered in the thermodynamics context, their additional validity in an information theoretical context allows one to alter the perfect mechanic of the Carnot cycle. For the isothermal phases, if mutual information between the two entropic, final and beginning, states exist, this alters the entropy availability and; accordingly, less free-energy is yielded. For the adiabatic phases, it seems that internal conversion between energy and work is perturbed by the cost introduced by the code conversion. This phenomenon does not occur in normal thermodynamic cycles (where we deal with Avogadro number of states and unperturbed statistics), but could occur in micro-systems or bio-molecular systems, where a limited numbers of molecules as well as a limited amount of information are present, such as the emerging field of the molecular information and the theory of information applied to living systems. The emergence of these effects introduces asymmetries in the cycle (elements of irreversibility) that the information theoretical methods could help to identify and characterize. The consequences of these effects are already emerging in some fields (see as example [14] and [17]) and others will predictably emerge.
